# Estimation of genotype-by-milking system interaction for daily milk yield in Holstein cattle

**DOI:** 10.3168/jdsc.2026-1077

**Published:** 2026-07-27

**Authors:** Patrick R. Fotso-Kenmogne, Paulo L.S. Carneiro, Delvan A. Silva, Musa Lamido, Carlos H.M. Malhado, Gilberto R.O. Menezes, Luiz F. Brito

**Affiliations:** 1 Postgraduate Program in Animal Science, State University of Southwest Bahia, Itapetinga, 45700-000, BA, Brazil; 2 Ministry of Employment and Vocational Training (MINEFOP), Yaoundé, P.O. Box 16273, CE, Cameroon; 3 Embrapa Beef Cattle, Brazilian Agricultural Research Corporation, Campo Grande, 79106-550, MS, Brazil; 4 Department of Biology, State University of Southwest Bahia, Jequié, 45206-190, BA, Brazil; 5 Department of Animal Sciences, Federal University of Viçosa, Viçosa, 36570-000, MG, Brazil; 6 Department of Animal Sciences, Federal University of Dustin-Ma, Katsina State, PMB 5001, Nigeria; 7 Department of Animal Sciences, Purdue University, West Lafayette, IN 47907

## Abstract

Milk production recorded by different milking systems is commonly combined for genetic evaluations. However, genotype-by-milking system interactions could occur for daily milk yield and these could affect animal ranking and the effectiveness of dairy cattle breeding programs. Therefore, we estimated (co)variance components and genetic parameters for daily milk yield (DMY) measured by milking robots or conventional milking parlors, considered as different traits, based on a 2-trait repeatability model. The systematic effects fitted in the model included calving year and season, DIM as a linear covariate, and calving age as linear and quadratic covariates. The random effects included additive genetic, permanent environment, and residual effects. Phenotypic records of 11,788 genotyped cows with 60,277 SNPs (after quality control) were used for the analyses. The traits have substantial additive genetic variance and low-to-moderate heritability estimates (from 0.10 ± 0.01 to 0.14 ± 0.01 for DMY measured by milking parlors, and from 0.20 ± 0.02 to 0.21 ± 0.02 for DMY from milking robots). The genetic correlation estimates (± posterior SD) for DMY based on milking system (milking robots × milking parlors) were 0.88 ± 0.03 in parity 1 and 0.92 ± 0.05 in parity 2. No evidence of strong genotype-by-milking system interaction was found between DMY measured by milking parlors or milking robots, indicating no differential responses to selection for DMY across milking systems.

## Graphical Abstract

**Figure:**
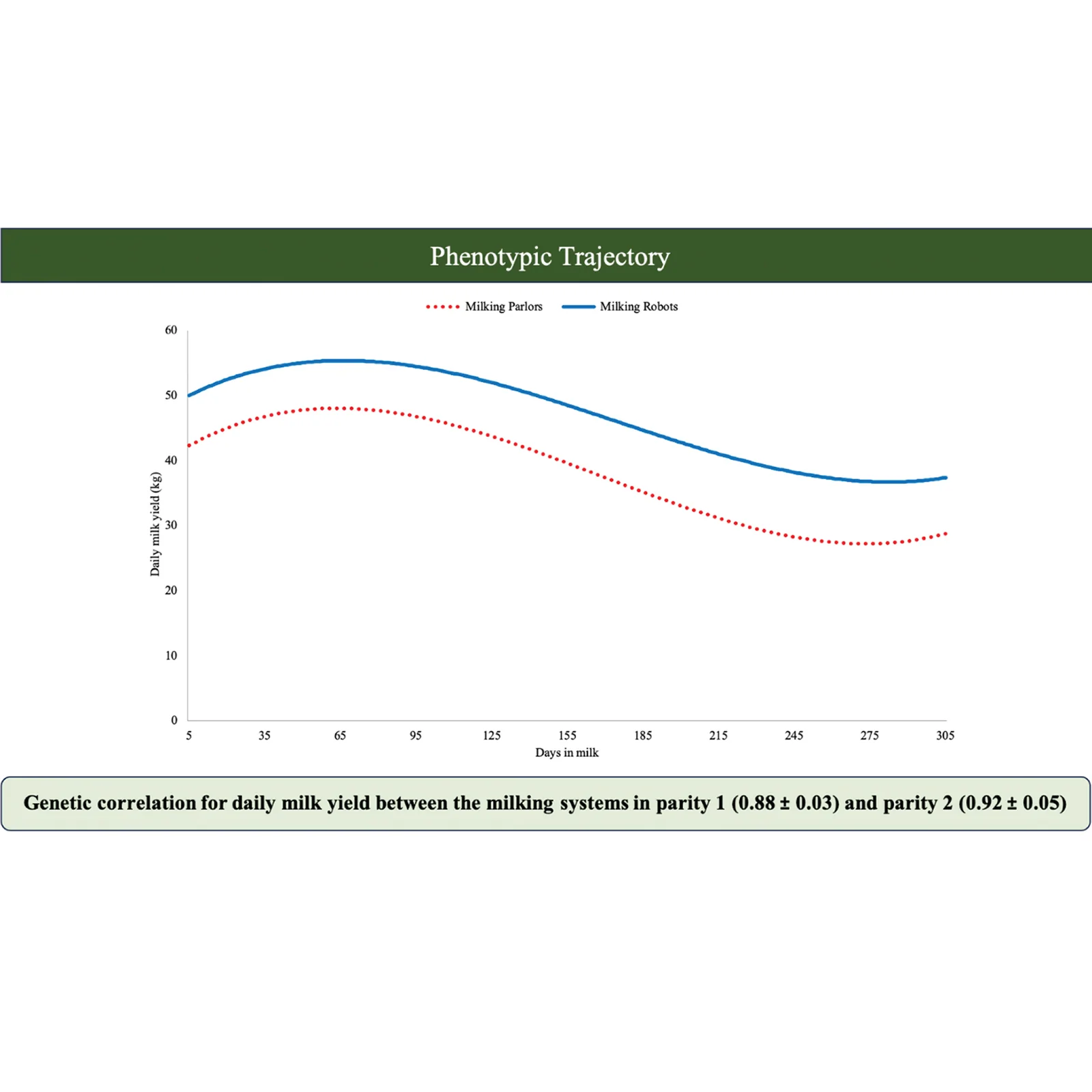

The dairy industry has experienced substantial changes for many decades (Barkema et al., 2015). The growing demand for high-quality food (e.g., milk) and advances in genetic selection, milking systems, nutrition strategies, and management practices have reshaped the present-day dairy sector (Lovarelli et al., 2020; Medeiros et al., 2022). The wide adoption of automated milking systems (**AMS**; also known as milking robots) is changing dairy production in many countries, providing a viable response to labor scarcity and at the same time facilitating improvements in milk hygiene, animal welfare, and the psychosocial well-being of farm operators (Lovarelli et al., 2020; Hunt, 2025). The adoption of AMS has enabled the collection of vast quantities of data regarding both overall production and individual animal health (Jacobs and Siegford, 2012; Pedrosa et al., 2023; Brito et al., 2025). Proper milking management is crucial for maximizing milk yield and flow while maintaining high hygiene standards and supporting the long-term health of the mammary system of cows (Rasmussen and Reinemann, 2010).

Automated milking systems are mainly designed to replicate standard manual milking protocols, ensuring that both pre- and postmilking procedures are in accordance with established best practices. Automated milking systems contribute to reducing the labor associated with milking, increase milk production, improve dairy cow welfare (Samoré et al., 2011; Brasier et al., 2025), and generate a large volume of phenotypic records, such as daily milk yield (**DMY**), milk electrical conductivity (Maskal et al., 2026), milking efficiency (Pedrosa et al., 2023), and cow behavior (Bérat et al., 2025). Increases in milk yield of 5% to 15%, as the result of an increase in daily milking frequency, have been reported when cows are milked by AMS (Rotz et al., 2003; Jacobs and Siegford, 2012). Hence, the success of AMS depends heavily on cow traffic (i.e., how easily cows can move through the AMS units). Additionally, conventional milking parlors (**MP**) continue to be used in large-scale operations because they offer greater input efficiency and lower capital investment per cow in large herds (Davies et al., 2010). Some studies have found that cows milked by MP tend to have lower SCC and better casein indices compared with those in AMS (Crocco et al., 2025). In MP, cows are milked in groups at specific times (usually twice or 3 times a day) by human operators in designated buildings (Jacobs and Siegford, 2012). Despite its lower cost than AMS, MP has some disadvantages, including higher labor demand and limited flexibility of milking schedule and frequency, which can affect milking management and cow productivity.

The differences between MP and AMS (Jacobs and Siegford, 2012) indicate that there could be genotype-by-milking system interactions, with potential implications for dairy cattle genetic evaluations. Behavioral responses in AMS are influenced by the animal's environmental conditions and their genetic background (Bérat et al., 2025). Therefore, the cows' performance may differ across environments. From a genetic selection perspective, there is a need to assess whether animals that are genetically selected to perform well in MP environments are also the best performers under AMS conditions, and vice versa. This concept can be referred to as genotype-by-environment interactions (**G×E**), which have been commonly reported in cattle populations (Silva Neto et al., 2024; Santana et al., 2025). Genotype-by-environment interactions can be quantified based on multitrait models, especially when environmental conditions can be categorized into a few distinct groups (e.g., milk yield recorded by AMS or MP). National genetic evaluations in dairy cattle have assumed that no genotype-by-milking system interaction exists. Therefore, the main objective of this study was to investigate the level of G×E for DMY in different milking systems (AMS and MP) in a United States Holstein cattle population.

The data used in this study were sourced from a large commercial dairy cattle farm located at north-central Indiana (USA) from 2014 to 2021. The datasets were gathered and integrated through the Purdue Animal Sciences Data Ecosystem (Boerman et al., 2025). The phenotypic and genomic datasets used were described in our previous study (Fotso-Kenmogne et al., 2026). Overall, the initial dataset consisted of 7,083,609 DMY records obtained from 29,343 lactations representing 14,469 animals managed using either AMS or MP. Data editing processes were implemented to improve data quality. Records were removed if they exhibited biologically unrealistic values, insufficient number of observations representing the lactation (<30 DMY records or lactation length <70 d), animals in their third and subsequent lactations, animals with age at first calving <640 d, and contemporary groups (**CG**) with fewer than 10 animals. Only DMY records within the range of 5 to 305 DIM were retained, and values deviating by more than 3.5 phenotypic SD from the CG mean were excluded from further analyses. Contemporary groups were defined by concatenating calving year and season. A total of 4,397,493 records from 17,874 lactations of 11,788 animals remained for further analyses, including 2,754,840 DMY of 11,139 cows in parity 1; and 1,642,653 DMY records of 6,735 cows in parity 2.

The genomic dataset was imputed from various SNP panels and contained 72,820 SNPs for 22,090 animals (Chen et al., 2023). The quality control procedure removed SNPs and animals with a call rate <0.90, SNPs with minor allele frequency <0.01, duplicated samples or SNPs, and SNPs with extreme deviation from the Hardy–Weinberg equilibrium (*P* < 10^−8^). The final genomic dataset contained 60,277 SNPs and 14,464 animals. Genotyped animals without phenotypic records were removed from subsequent analyses.

A 2-trait repeatability model was used to perform the analyses. Repeatability models provide a more simplified treatment of repeated daily records, assuming constant within-cow covariance across lactation. The model included the systematic effects of CG, DIM as a linear covariate of DIM (5–305 d), calving age as linear and quadratic covariates, and the random effects of additive genetic, permanent environment, and residual. The model can be described as follows:

**y** = **Xb** + **Za** + **Wpe** + **e**,

where **y** represents the vector of phenotypic records for each trait (DMY in an AMS [**DMY_AMS**] or in an MP [**DMY_MP**]) in parities 1 and 2; **b** is the vector of systematic effects, as described previously; **a** is the vector of random additive genetic effects with $a∼N\left(0,G\;⊗\;\Sigma_{a}\right),$ where **G** represents the genomic relationship matrix calculated based on the first method proposed by VanRaden (2008) and **Σ_a_** is the covariance matrix for the additive genetic effects; **pe** is the vector of random permanent environmental effects with $pe∼N\left(0,\;I_{P}\;⊗\;\Sigma_{P}\right),$ where **I_p_** represents an identity matrix and **Σ_p_** is the covariance matrix associated with the permanent environmental effects; **e** is the vector containing the random residual matrix effects with $e∼N\left(0,\;I\;⊗\;\Sigma_{R}\right),$ where **I** represents an identity matrix, **Σ_R_** is the residual covariance matrix, and ⊗ denotes the Kronecker product. **X**, **Z**, and **W** are the incidence matrices relating **b**, **a**, and **pe** to **y**. The estimation of (co)variance components was based on Bayesian inference using the Gibbs sampler algorithm implemented in the GIBBSF90+ version 3.19 software from the BLUPF90 family of programs (Misztal et al., 2018). The threshold for genetic correlations used to indicate the presence of strong G×E was 0.80, with values below this threshold indicating a significant G×E effect (Robertson, 1959).

Phenotypic averages estimated across different milking system types (AMS and MP) for DMY in parities 1 and 2 according to DIM are illustrated in Figure 1. The phenotypic curve patterns were similar in parity 1 (Figure 1a) and parity 2 (Figure 1b). However, the average DMY was consistently higher in AMS compared with MP throughout the entire lactation period. This is likely the result of management practices because the farmers usually assign cows of higher milk production level to AMS and an increase in milk yield may also result from the greater milking frequency in AMS (the average number of visits per day in the AMS was 3.65 ± 1.97; Bérat et al., 2025). Different trends in average DMY over DIM were observed based on the milking system type (higher DMY_AMS than DMY_MP). These differences can be attributed to the frequency of daily visits (i.e., milking frequency). Furthermore, the increase in milking frequency is related to improved animal welfare, and consequently greater performance (Rotz et al., 2003; Mulder et al., 2004).

**Figure 1:**
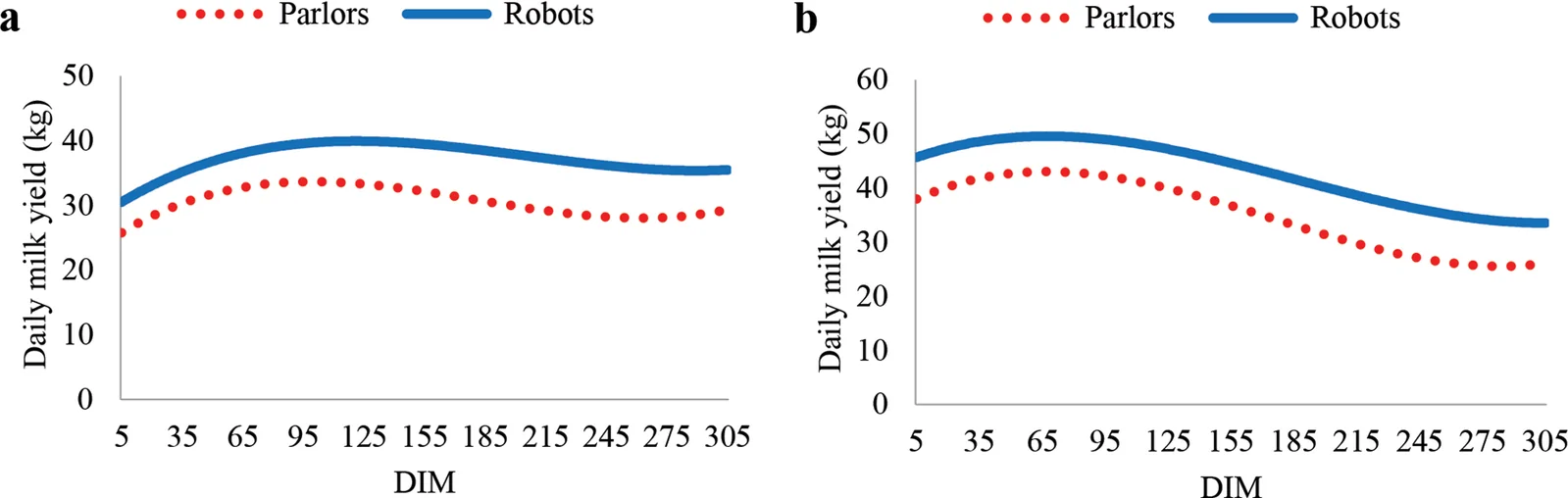
Phenotypic average estimated across different milking type (i.e., MP and AMS [milking robots]) for DMY in American Holstein cattle according to DIM in parities 1 (a) and 2 (b).

Variance components, heritability, repeatability, and genetic correlation for DMY in parity 1 and parity 2 are presented in Table 1. The permanent environmental variance (± posterior SD) ranged from 14.38 ± 0.28 for MP to 23.49 ± 0.92 for AMS in parity 1, and from 25.34 ± 0.71 for MP to 30.73 ± 1.63 AMS in parity 2. In both parities (1 and 2), the heritability estimates were low to moderate for DMY_MP and DMY_AMS, respectively, indicating that genetic progress for these traits is feasible. Repeatability estimates for DMY_AMS were 0.62 ± 0.01 and 0.57 ± 0.01 in parities 1 and 2, respectively, whereas corresponding estimates for DMY_MP were 0.40 ± 0.01 and 0.37 ± 0.01, respectively (Table 1). Repeatability values between 0.30 and 0.60 are considered to be moderate, suggesting that these traits remain relatively stable and consistent across milking systems. The genetic correlations between DMY_MP and DMY_AMS were 0.88 ± 0.03 in parity 1 and 0.92 ± 0.05 in parity 2, respectively, indicating no evidence of strong G×E for DMY across milking system types.

**Table 1:** Posterior means (SD) of variance components, heritability, repeatability, and genetic correlation (AMS × MP) for DMY in American Holstein cattle^1^

Variable	Parity 1		Parity 2	
	MP	AMS	MP	AMS
Additive genetic variance	7.933 (0.470)	12.088 (1.125)	9.052 (0.870)	16.933 (2.254)
Permanent environmental variance	14.378 (0.281)	23.485 (0.920)	25.335 (0.707)	30.732 (1.631)
Residual variance	33.948 (0.032)	21.433 (0.045)	57.805 (0.077)	35.953 (0.074)
Heritability	0.141 (0.007)	0.208 (0.017)	0.098 (0.008)	0.202 (0.024)
Repeatability	0.397 (0.005)	0.624 (0.008)	0.373 (0.006)	0.570 (0.009)
Genetic correlation (AMS × MP)	0.875 (0.031)		0.919 (0.054)	

1 AMS = automatic milking system; MP = milking parlor.

In general, additive genetic variances were higher for DMY_AMS than DMY_MP, suggesting a greater variability, probably due to differences in milking frequency. Genetic variance is essential for a population and is crucial for selection. The population analyzed in this study consisted of animals from a single large commercial dairy farm in the United States (Pedrosa et al., 2023; Fotso-Kenmogne et al., 2025), which can contribute to minimizing environmental variation across the milking systems. Therefore, it is important to carefully monitor the genetic variance in populations to support their management practices. The performance of high-producing dairy cows is strongly dependent on the interaction between their genetic background and the environmental conditions. The fact that farmers usually assign cows of higher milk production level to AMS could lead to a bias in environment categorization (due to pre-existing genetic merit), potentially influencing variance components estimates and therefore heritability estimates.

The residual variance decreased with AMS. In contrast, Mulder et al. (2004) who investigated G×E between AMS farms and conventional milking farms in Dutch Holstein cows using multivariate fixed regression test-day sire models, reported an increase in residual variance with AMS. This difference may be attributed to variations in the sampled population, data structure, model, and the relationship matrix used. In this study, DMY records and genomic information were used, which allow for more accurate estimates (Pedrosa et al., 2023). Unlike previous studies (Mulder et al., 2004; Carlström et al., 2014) that were based on test-day records and used pedigree-based relationship matrices, this study employed DMY records together with a genomic relationship matrix. The use of daily measurements reduces potential bias arising from nondaily data collection. The genetic correlations between DMY_AMS and DMY_MP observed in this study were high (>0.80), indicating no potential differential genetic responses across milking systems. Carlström et al. (2014) estimated genetic correlations for different milkability traits across milking systems (i.e., AMS and MP). The genetic correlations reported were close to unity, especially for average flow rate (0.97–0.98) and milking time (0.98–1.00). The authors concluded that the high genetic correlations between milking systems (AMS and MP) support the joint use of data from both systems in the genetic evaluation of milkability. Mulder et al. (2004) reported a genetic correlation of 0.89 for test-day milk yield between AMS farms and conventional MP farms, indicating that G×E effects are small; therefore, AMS farms can accurately select animals based on genetic evaluations that combine data from both milking systems.

The differences in heritabilities between DMY_AMS and DMY_MP were considerable. The lower heritability estimate for DMY_MP observed in parity 2 may be attributed to selection decisions and culling practices because cows are selected based on their previous performance. Management of cows across milking systems could also influence these estimates because the higher performing cows in the MP may be moved to the AMS. To date, genetic responses of DMY across milking system types using genomic information do not appear to have been reported in the literature. Therefore, a next step will be to investigate genotype-by-milking system interaction for milk composition traits and lactation persistency using large datasets from multiple populations. Given that cows in the AMS system were milked more frequently, this could have influenced the differences in DMY observed. Therefore, milking frequency should be included in the model in future studies if it is found to significantly influence phenotypic performance, particularly when there are large differences in the number of daily milkings in MP, which was not the case in the present study.

In conclusion, we evaluated genotype-by-milking system interaction (AMS × MP) for DMY in American Holstein cattle based on 2-trait repeatability model. High and positive genetic correlations between DMY_AMS and DMY_MP were observed, indicating that no evidence existed for a strong genotype-by-milking system interaction for DMY across milking systems.
